# A better model for care — virtual care coordination

**Published:** 2012-06-15

**Authors:** Chris Herbert, Claire Medd

**Affiliations:** Care Innovations, UK; Care Innovations, UK

**Keywords:** care innovations, virtual care coordination, comprehensive care model, chronic conditions

## Abstract

Telehealth is a proven tool for reducing costs, improving quality of life, reducing hospital visits/length of stay and enhancing relationships between patients and community nurses. There have been many trials demonstrating these benefits, and findings have always demonstrated return on investment. However, uptake has been slow—68% of providers have no plans to roll out a telehealth solution in the next 24 months. Through a decade of ethnographic research, Care Innovations has visited 20 countries, 1000 homes and 150 hospitals and clinics, generating more than a dozen pilot projects in homes and a number of research projects, to provide a better understanding of where telehealth fits within care models and how to maximise the benefits delivered by improving technologies.

Rather than a stand-alone device that provides medical readings remotely, clinicians and patients require a system-level, standards-based approach that is adaptable to practical healthcare system needs—from assessing patient status to providing better management of chronic conditions to compatibility with medical records systems. In this patient-centred approach, many factors, including psychosocial ones, are important to the process. Things like fostering an alliance between a patient and clinicians, reinforcing a patient’s desires and life goals, using more effective messages and positive psychology to motivate a patient to make difficult lifestyle changes, and personalising each course of treatment become integral to the process.

Virtual care coordination establishes an active and dynamic support network, linking patients with clinicians and doctors. Using computer-based biometric monitoring, two-way videoconferencing, educational tools, and logic-based evaluation techniques, patients gain more control over the management of their chronic conditions while being comfortable within their home environment. This approach helps provide quality, cost effective treatment of long-term diseases, encouraging healthy behaviours and providing positive feedback to guide the health process. Treating the whole person in this manner is an emerging field referred to as integrative care.

Integrative care via virtual care coordination is a model that encompasses a variety of disciplines and that treats the whole person, not simply the disease. Using the strengths of conventional medicine combined with complementary treatments (that might include biofeedback, meditation, dietary changes, and stress reduction techniques), integrative care establishes a deeper relationship between the patient and the physician, emphasising wellness and patient self-care. Four elements combine to assist patients in making the necessary lifestyle changes to successfully improve their health and to manage chronic conditions more effectively:

- Engage: Make interaction with the device a pleasant, personalised experience to encourage long-term use and acceptance.
- Educate: Give patients access to relevant, timely information through videos and instructional material to support lifestyle changes and the prescribed treatment plan.
- Evaluate: Provide a way for physicians and clinicians to remotely assess improvements or detect setbacks in a patient’s condition and respond quickly when necessary.
- Empower: Give patients the means to manage their conditions actively and to live as independently as possible.

Drawing on findings and insights from recent pilot studies that demonstrate the successful use of virtual care coordination, Claire Medd will highlight key outcomes, demonstrating how telehealth can be successfully incorporated into a comprehensive care model to increase the value and outcomes of pilots and rollouts.

